# Poster Session I - A171 OUTCOMES OF ENDOSCOPIC MUCOSAL RESECTION OF LARGE COLORECTAL POLYPS REFERRED TO A SINGLE TERTIARY CENTRE

**DOI:** 10.1093/jcag/gwaf042.171

**Published:** 2026-02-13

**Authors:** N Ashrafinia, N Umar, T Issa, R Sultanian, S Zepeda Gomez

**Affiliations:** University of Alberta Faculty of Medicine & Dentistry, Edmonton, AB, Canada; University of Alberta Faculty of Medicine & Dentistry, Edmonton, AB, Canada; University of Alberta Faculty of Medicine & Dentistry, Edmonton, AB, Canada; University of Alberta Faculty of Medicine & Dentistry, Edmonton, AB, Canada; University of Alberta Faculty of Medicine & Dentistry, Edmonton, AB, Canada

## Abstract

**Background:**

Endoscopic mucosal resection (EMR) is widely used for removing large colorectal polyps (≥20mm). The goal is complete resection with minimal adverse events and a low adenoma recurrence rate (ARR). Optimal initial evaluation of the polyps and subsequent resection by a therapeutic endoscopist is of crucial importance to achieve good outcomes.

**Aims:**

To evaluate the accuracy of the initial evaluation of large polyps referred for EMR to a tertiary Centre. Subsequently, we analyzed the EMR technical success, complications, recurrence rate, final histology, appropriate follow-up, and need for surgery for patients referred for EMR of large colonic polyps.

**Methods:**

This was a retrospective review of prospectively collected data of patients referred for EMR of colorectal polyps ≥20mm between 2017 and 2024. The procedures were performed by two therapeutic endoscopists.

**Results:**

A total of 170 patients (median age 66; 52.3% female) with 187 polyps (median size 30mm) were included. Among referred polyps, 89 (48%) had accurate descriptions; 50 (27%) underestimated size, 41 (22%) overestimated size, 16 (8.6%) misclassified Paris type, and the location description was not accurate in 9 (5%). Initial manipulation of polyps at the first endoscopy was done in 58% of polyps; this included biopsies of the polyp (6.4%), tattooing nearby (8.4%), lifting (7.5%), and attempted resection in 15.7%. Median time to first EMR was 30 days. Median polyp size was 30mm (range 20-80mm). After EMR, high-grade dysplasia (HGD) was reported in 28 polyps (15%) and invasive carcinoma in 7 (3.7%). EMR could not be completed in 2 cases (1.1%) due to suspicion of submucosal invasion. Complications included mild intra-procedural bleeding in 37 patients (20%), post-polypectomy bleeding in 3 (1.7%), perforation in 1 (0.5%), which was managed conservatively and one case of post-polypectomy syndrome. Average time to follow-up was 7 months. Residual adenomatous tissue was present in 33 cases (18%), with HGD in 9 (5%). ARR increased according to polyp size (11.5% for 20-29mm, 13.5% for 30-39mm, and 29.0% for ≥40mm).

**Conclusions:**

Initial polyp description was inaccurate in around 50% of cases before referral for EMR and around 58% of polyps had some form of previous endoscopic manipulation as well. EMR is a safe and effective treatment for large colorectal polyps when performed by expert endoscopists. Subsequent follow-up met recommended guidelines and ARR correlated significantly with polyp size.

**Funding Agencies:**

None

